# Editorial Notes

**Published:** 1884-02

**Authors:** 


### Editorial Notes.

We call attention of our readers to the value of nitrogenized iron, prepared by Chapman, Green & Co., of Chicago. It is by far the most palatable of all the preparations of iron. We have used it extensively at the Buffalo Medical Dispensary and in private practice, with most excellent results.

Drs. Hopkins and Mann represented the Committee on Legislation of Erie county before the Committee of the Legislature, to whom the bill was referred. Drs. Vanderveer, Sturgis, St. John Roosa and Lawrence Johnson were also at the hearing given by the committee to the advocates of the bill.

Dr. J. S. Jewell, Editor *Journal of Mental and Nervous Diseases*, and Professor of Mental and Nervous Diseases, Chicago Medical College, Chicago, Ill., says: "I have used Battle & Co.'s preparation known as Bromidia, and believe it to be as reliable as it is represented to be by its proprietors. I have thus far been pleased with its effects."

W. H. Tibbs is one of our most reliable and trustworthy druggists. Any promises he may make will be fulfilled. It is therefore worth while to any physician desiring to purchase surgical instruments, to look over his large stock, which he advertises for sale at a sacrifice to close out stock. It is an unusual chance to buy ininstruments at a low price.

C. H. Hughes, M. D., Lecturer on Psychiatry and Neurology, Post-Graduate Faculty, St. Louis Medical College, Editor *Alienist and Neurologist*, St. Louis, Mo., says: "I frequently prescribe Celerina when I want to use a reliable compound of celery and coca, and the prescription has given me satisfaction in its results as a nerve-tonic in many cases."

We call attention to the letter of Dr. Albert L. Gihon, President of the American Public Health Association, Vice-President American Medical Association, Medical Director United States Navy, etc., as another illustration of the sentiment of the best men in the profession on the subject of the State Faculty Bill. This is but one of the many letters constantly being received by the committee from distinguished men in hearty support of this measure.

THE attention of our readers is called to the original communication on dysentery, constituting the leading article in this number of the JOURNAL, as one well worthy of their perusal and favorable opinion. While expressing this approving criticism, we would yet call attention to the fact that much remains to be said, and we invite those who have anything to say, and who will do so briefly, to send us communications for publication in subsequent numbers.

THE prospects for the success of the State Faculty Bill are brighter than at any time since the initiative was taken by the Erie County Medical Society. It is a matter for profound regret to all the real friends of the oldest medical college in Buffalo, that its faculty should not have bravely led the van with the County Society in the battle for the advancement of the best interests of profession and people. No little credit is due, however, to that member of its faculty who has been a member of the Committee on Legislation and has worked with energy for the success of the bill.

WE have received, through Prof. George E. Fell, of the Committee on Publication, a copy of the proceedings of the American Society of Microscopists, at its sixth annual meeting, held in Chicago, August 7, 8, 9, 10, 1883. The volume comprises 275 pages of matter, consisting of papers from eminent microscopists of this country and a list of members of the society. The paper and typography, and its general appearance, are highly creditable to the Publication Committee, and also to Messrs. Haas & Klein, of this city, from whose establishment it was issued. The society, of whose work the present volume is an exponent, is the only organization of its kind, either here or abroad, which devotes its labors exclusively to subjects wherein the microscope is the essential agency or instrument in investigation and study. The work, therefore, is all the more creditable, and places our scientists in the front rank of earnest investigators. We regard the society and its labors with great pride and favor, and trust the medical profession will continue to encourage earnest work in this important field, and to study the results, which, in skillful hands, the microscope is bringing forth.

